# Is ^18^F-fluoride PET/CT an Accurate Tool to Diagnose Loosening After Total Joint Arthroplasty?

**DOI:** 10.1097/CORR.0000000000003228

**Published:** 2024-09-11

**Authors:** Caroline Sköld, Jens Sörensen, Anders Brüggemann, Nils P. Hailer

**Affiliations:** 1 Department of Surgical Sciences/Orthopaedics, Uppsala University, Uppsala, Sweden; 2 Department of Surgical Sciences/Nuclear Medicine and PET, Uppsala University, Uppsala, Sweden

## Abstract

**Background:**

Several studies using positron emission tomography (PET) show highly elevated periprosthetic bone uptake of fluorine-18 sodium fluoride (^18^F-fluoride), suggestive of implant loosening after arthroplasty. Focus so far has been on qualitative but not on quantitative assessment. There is also a lack of intraoperative confirmation of preoperative ^18^F-fluoride PET findings. Although the method seems to have acceptable accuracy and high sensitivity, an attempt to improve the specificity and an overall validation of the method appear warranted.

**Questions/purposes:**

(1) Is there a difference in ^18^F-fluoride uptake around loose versus well-fixed THA and TKA components? (2) Can ^18^F-fluoride uptake measures provide a threshold that differentiates loose from well-fixed implants undergoing revision for a variety of septic and aseptic indications? (3) In a population restricted to THA and TKA undergoing revision for aseptic indications, can measurement of ^18^F-fluoride uptake still distinguish loose from well-fixed components? (4) What is the interrater reliability of measuring ^18^F-fluoride uptake?

**Methods:**

This was a retrospective assessment of a diagnostic test, ^18^F-fluoride PET/CT, which was performed prior to revision surgery. We included 63 patients with 31 THAs and 32 TKAs. Sixty-five percent of patients were female, and the mean age at ^18^F-fluoride PET/CT was 66 years. The THA had different modes of fixation (cemented, cementless, and hybrid; 45%, 32%, and 23%, respectively), whereas all TKAs were cemented. Imaging was conducted using routine protocols 1 hour after tracer injection. The interobserver reproducibility was analyzed using Spearman rank correlations and Bland-Altman analyses. Two independent observers were trained separately by a nuclear physician to measure maximal periprosthetic standardized uptake values (SUV_max_) for each arthroplasty component (n = 126). Findings at surgery (whether the components were well fixed or loose, as well as the presence or absence of infection) were used as a reference. Presence of periprosthetic joint infection was retrospectively determined based on the criteria suggested by the European Bone and Joint Infection Society (EBJIS): clinical features in combination with blood analysis, synovial fluid cytologic analysis, and microbiology test results. Receiver operating characteristic (ROC) curves were plotted to assess the area under the curve (AUC) for each investigated component separately, indicating suitable SUV_max_ thresholds that differentiate loose from well-fixed components. After excluding patients with confirmed or suspected PJI per the EBJIS criteria (n = 12), the above analysis was repeated for the remaining patients with aseptic loosening (n = 51).

**Results:**

We found higher ^18^F-fluoride uptake around loose versus well-fixed components in all but femoral TKA components (median [range] SUV_max_ for well-fixed versus loose THA cups 10 [7 to 30] versus 22 [6 to 64], difference of medians 12; p = 0.003; well-fixed versus loose TKA femoral components 14 [4 to 41] versus 19 [9 to 42], difference of medians 5; p = 0.38). We identified favorable ROC curves for all investigated components except femoral TKA components (THA cups AUC 0.81 [best threshold 13.9]; THA femoral stems AUC 0.9 [best threshold 17.3]; femoral TKA components AUC 0.6 [best threshold 14.3]; tibial TKA components AUC 0.83 [best threshold 15.8]). ^18^F-fluoride was even more accurate at diagnosing loosening when we limited the population to those patients believed not to have prosthetic joint infection (THA cups AUC 0.87 [best threshold 14.2]; THA femoral stems AUC 0.93 [best threshold 15.0]; femoral TKA components AUC 0.65 [best threshold 15.8]; tibial TKA components AUC 0.86 [best threshold 14.7]). We found strong interrater correlation when assessing SUV_max_ values, with Spearman ρ values ranging from 0.96 to 0.99 and Bland-Altman plots indicating excellent agreement between the two independent observers.

**Conclusion:**

Measuring SUV_max_ after ^18^F-fluoride PET/CT is a useful adjunct in the diagnostic evaluation for suspected implant loosening after THA and TKA. The method appears to be both accurate and reliable in diagnosing implant loosening for all components except femoral TKA components. In a real-world mixed population with both low-grade infection and aseptic loosening, the method seems to be fairly easy to learn and helpful to subspecialized arthroplasty clinicians. When infection can be ruled out, the method probably performs even better. Further prospective studies are warranted to explore the reason why femoral TKA component loosening was more difficult to ascertain using this novel technique.

**Level of Evidence:**

Level III, diagnostic study.

## Introduction

Aseptic loosening remains the leading cause of revision surgery after THA and TKA [5, 13]. In Sweden, roughly 3500 THA and TKA revisions were performed in 2022. The cause for revision differs over time, with periprosthetic joint infection (PJI) being more common early postoperatively; but overall, the leading cause for THA revision was aseptic loosening (41.6%), followed by infection (20.8%), periprosthetic fracture (15.7%), and instability (12.2%) in the Swedish Arthroplasty Register [31]. Implant loosening, either due to septic or aseptic reasons, causes osteolysis and is strongly associated with an increase in periprosthetic bone metabolism [11, 24]. This process is characterized by activation of both osteoblasts and osteoclasts on a cellular level [10, 20, 24, 26]. The enhanced osteoblastic activity has traditionally been visualized by bone scintigraphy after injection of technetium-99m (99mTc), a test that, although sensitive, is lacking in specificity and has low spatial resolution. Mumme et al. [23] found a sensitivity of 78%, specificity of 70%, and overall accuracy of 74% for this method, and a pooled sensitivity of 85% and specificity of 72% was reported in a large meta-analysis [32].

Indium-labeled white blood cell (WBC) imaging has been developed as an alternative, but the main aim of this method has been to detect and identify PJI [25].

Because of the limitation of 99mTc bone scans and indium-labeled WBC scans, newer techniques in nuclear medicine have assessed the utility of positron emission tomography (PET) combined with CT using the fluorine-18 sodium fluoride isotope (^18^F-fluoride PET/CT) in the diagnosis of implant loosening. Fluoride (F) ions exchange with hydroxyl groups of hydroxyapatite at the surface of bone crystals, forming fluorapatite at sites of bone remodeling. Therefore, the uptake of ^18^F mainly reflects local bone blood flow and osteoblastic activity [9]. ^18^F-fluoride PET/CT appears to have acceptable accuracy and high sensitivity but modest specificity [30]. In most studies ^18^F uptake patterns have been qualitatively assessed, with only a few reporting quantitative analyses of ^18^F-fluoride uptake [15-17, 30]. Moreover, there is a lack of intraoperative confirmation of preoperative ^18^F-fluoride PET/CT findings.

Blau et al. [4] were the first to use ^18^F-fluoride in bone imaging, and Creutzig [7], who investigated 31 hip prostheses using planar ^18^F-fluoride scanning, found that the nonspecific uptake tends to diminish between 6 and 9 months after surgery. ^18^F-fluoride PET with or without CT has since been used in the investigation of bone metabolism in osteoporosis [2, 28], femoral head necrosis [27, 35], bone-impaction grafting [3, 33, 34], renal osteodystrophy [22], and bone malignancies [1, 12]. A study by Sterner et al. [30], which examined 14 patients with painful TKA using diagnostic ^18^F-fluoride PET/CT, found excellent sensitivity but notably low specificity. In that study, only six patients were revised, and the findings were determined through qualitative analysis of tracer uptake.

Standardized uptake values (SUVs) are the most widely used index for comparing the intensity of PET tracer uptake between scans in the same individual or among different individuals. The SUV index normalizes uptake intensity for differences in injected dose and body weight and is widely used in oncologic PET imaging. Measuring maximal SUV (SUV_max_) values around implants should enable quantitative analysis of ^18^F uptake, potentially surpassing previous qualitative assessments. To date, no study has investigated the reliability and accuracy of measuring SUV_max_ using ^18^F-fluoride PET/CT to diagnose THA or TKA loosening. Given that ^18^F-fluoride uptake in the bone reflects periprosthetic increase in blood flow for various reasons, including infection, this can cause confusion in the interpretation of SUV_max_ in the context of implant loosening [6, 15].

We therefore asked: (1) Is there a difference in ^18^F-fluoride uptake around loose versus well-fixed THA and TKA components? (2) Can ^18^F-fluoride uptake measures provide a threshold that differentiates loose from well-fixed implants undergoing revision for a variety of septic and aseptic indications? (3) In a population restricted to THA and TKA undergoing revision for aseptic indications, can measurement of ^18^F-fluoride uptake still distinguish loose from well-fixed components? (4) What is the interrater reliability of measuring ^18^F-fluoride uptake?

## Patients and Methods

### Study Design and Setting

This study was a retrospective assessment of a diagnostic test, ^18^F-fluoride PET/CT, that included 63 patients scheduled for revision of THA (n = 31) or TKA (n = 32) between May 2016 and February 2021 at the Department of Orthopaedics and Hand Surgery, Uppsala University Hospital, Uppsala, Sweden.

### Patients

Between May 2016, when a new PET device was installed, and February 2021, we performed 689 revision arthroplasties at our department (510 revision THA and 179 revision TKA). During that period, we increasingly used ^18^F-fluoride PET/CT in the diagnostic evaluation of painful THA and TKA, and a total of 199 ^18^F-fluoride PET/CT examinations were performed in our unit. Of those, 121 were performed for suspected loosening, whether for mechanical or septic reasons. We then crossmatched that cohort with our local arthroplasty register for performed revision procedures and identified a total of 66 procedures eligible for this study. One THA was excluded due to severe motion artifacts between the PET and CT scan, one knee joint in a patient with bilateral TKA was excluded due to dependency issues associated with bilateral observations, and one THA was excluded due to a severe PJI with an intraosseous abscess near the acetabulum. In the last case, PET/CT was performed to exclude or identify multiple infection sites in other locations and other implants. This left 63 patients with as many implants in the final study cohort. In total, ^18^F-fluoride PET/CT was performed in 9.6% of all revision procedures performed at our unit (7% of THA; 18% of TKA) during this time span.

Each study participant presented with a painful THA or TKA, along with radiologic or clinical indications of either suspected aseptic loosening, or a clinical suspicion of PJI. Still being a non–evidence-based and expensive examination, ^18^F-fluoride PET/CT was primarily used in patients in whom diagnostic uncertainty after conventional evaluation remained; thus, all included patients underwent routine clinical examination, laboratory testing, and radiographic imaging in addition to ^18^F-fluoride PET/CT before their subsequent revision procedure.

### Descriptive Data

The final cohort included a slight majority of female patients (65% [41 of 63]), as did the groups of patients with THA (61% [19 of 31]) and those with TKA (69% [22 of 32]). The mean age at ^18^F-fluoride PET/CT was 66 years (67 for patients with THA, 66 for those with TKA). The modes of THA fixation were either cemented in 45%, cementless in 32%, or hybrid in 23%, whereas all TKA were cemented. The time span between the index arthroplasty and investigation by ^18^F-fluoride PET/CT ranged from < 1 year (two patients) to 23 years. The time span between ^18^F-fluoride PET/CT and revision surgery varied between 1 month and 3 years (Table 1).

**Table 1.** Baseline patient characteristics

Characteristic	Hip (n = 31)	Knee (n = 32)	Overall (n = 63)
Sex			
Female	61 (19)	69 (22)	65 (41)
Male	39 (12)	31 (10)	35 (22)
Age at ^18^F-fluoride PET/CT	67 (42-82)	66 (43-82)	66 (42-82)
Fixation of revised component			
Cemented	45 (14)	100 (32)	73 (46)
Cementless	32 (10)	0 (0)	16 (10)
Hybrid	23 (7)	0 (0)	11 (7)
Presence of PJI (EBJIS classification)			
0	84 (26)	78 (25)	81 (51)
1	10 (3)	0 (0)	5 (3)
2	6 (2)	22 (7)	14 (9)
Time from original surgery to ^18^F-fluoride PET/CT in years	7.8 (0.8-23)	3.7 (0.6-17)	5.7 (0.6-23)
Time from ^18^F-fluoride PET/CT to revision surgery in months	7.5 (1-23)	9.4 (2-35)	8.5 (1-35)

Data presented as % (n) or mean (range); PJI = periprosthetic joint infection; EPJIS = European Bone and Joint Infection Society.

PJI was determined based on clinical features and blood test results, synovial fluid analysis, and microbiology test results. Retrospectively, each patient was classified as either unlikely to be infected, likely to be infected, or confirmed to be infected following the definition of PJI suggested by the European Bone and Joint Infection Society (EBJIS) [21]. This three-level classification has been endorsed by the EBJIS, the Musculoskeletal Infection Society, and the European Society of Clinical Microbiology and Infectious Diseases. In summary, the definition takes clinical features and diagnostic tests into account, and if all criteria are negative, infection is deemed unlikely. Two or more positive results indicate a likely infection, whereas the presence of a sinus tract, a positive alpha-defensin test result, or ≥ 2 positive microbiological cultures with the same pathogen confirm a PJI. The majority of patients in our cohort (81% [51 of 63]) were classified unlikely to be infected, a small number were classified as likely to be infected (5% [3 of 63]), and the remaining 14% (9 of 63) were classified as having confirmed PJI (Table 1). This evaluation was done over the entire follow-up period to catch the false-negatives.

### Surgical Procedures

Revision procedures were performed at the Arthroplasty Unit, Department of Orthopaedics and Hand Surgery, Uppsala University Hospital. Whether THA or TKA components were well fixed or not was intraoperatively assessed by the attending surgeon. Since ours is a retrospective study, no specific protocol regarding the intraoperative evaluation of implant fixation existed; instead, the surgical notes were assessed and interpreted by an experienced orthopaedic surgeon (CS).

### PET Scanning

Imaging was performed with a Discovery MI (GE) PET/CT scanner. A routine protocol was used in which 3 MBq of ^18^F-fluoride NaF per kilogram of bodyweight was injected intravenously 1 hour before scanning. Relevant body sections were imaged by PET, and a medium-dose CT was performed without iodine contrast media using the vendor’s image acquisition and reconstruction parameters. Images were analyzed and reported using the PET/CT tool in a picture archiving and communication system (PACS) (Philips).

### Image Interpretation

An experienced nuclear medicine physician (JS or other consultant nuclear physicians) visually evaluated the scans during routine diagnostics. The evaluation criteria were determined based on previously reported methods that focused on the pattern and location of NaF uptake at the interface of the prosthesis and bone or cement and bone [9, 30].

### SUV_max_ Measurement

Within the context of this study, two independent observers (CS and a medical student) further quantitatively evaluated the ^18^F-fluoride PET/CT results, and they assessed the SUV_max_ around the implants for each component (n = 126). The two observers’ introduction to PET imaging was conducted by an experienced nuclear medicine physician (JS) in a standardized way, teaching both observers how to use the measuring tools that are integrated in the commercially available Carestream PACS PET/CT viewer (Philips) that is routinely used at our institution. The introduction was completed in < 1 hour. All measuring points were documented using the “print screen” function and saved, ensuring that the nuclear medicine physician was able to reassess measurements whenever needed.

**Fig. 1:**
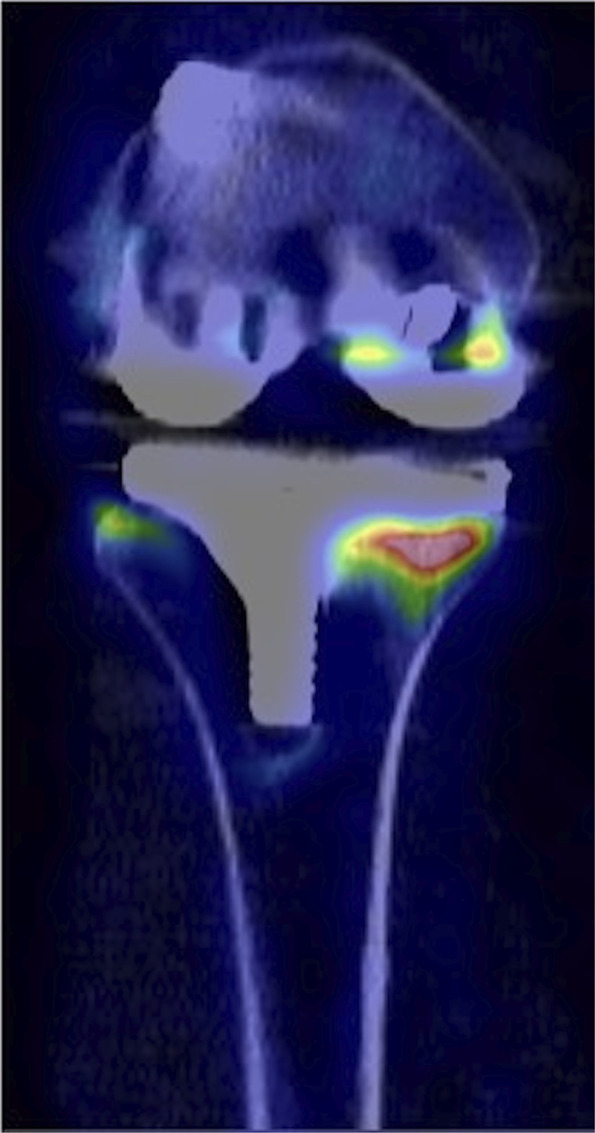
This image shows one of the TKAs enrolled in this study. The patient was female, 54 years of age, and presented at the clinic with instability and swelling. Evaluation was not conclusive. An ^18^F-fluoride PET/CT scan gave an SUV_max_ of 24 around the tibia and 16 at the femur. At revision surgery, the tibia was grossly loose and the femur was well fixed.

**Fig. 2:**
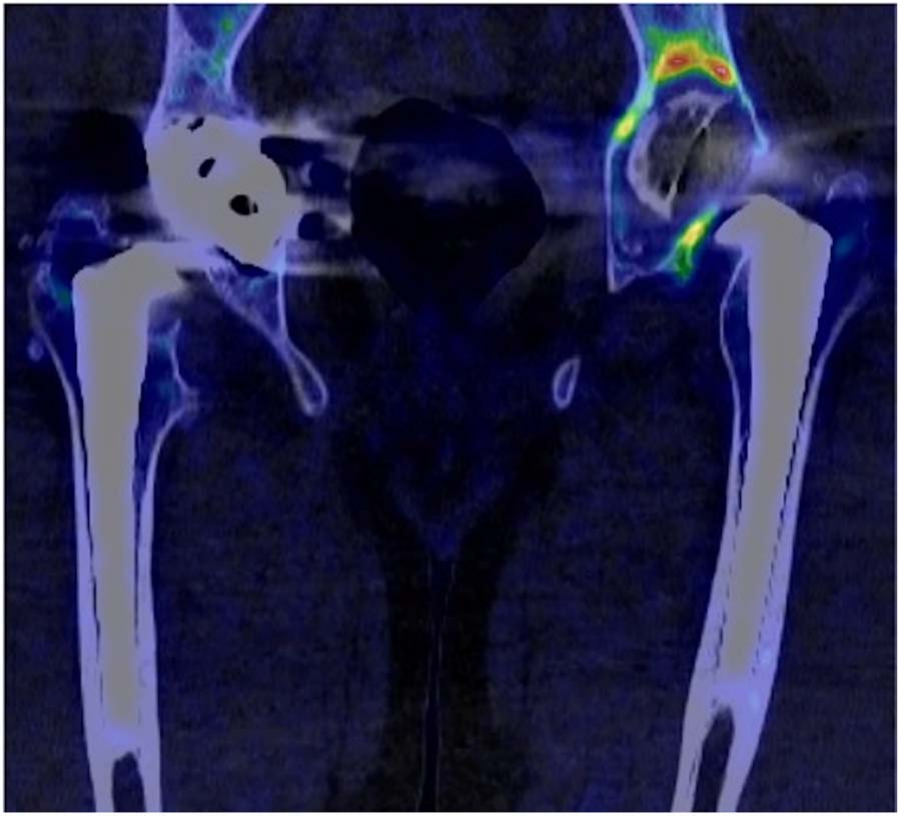
This image shows one of the THAs included in this study. The patient was male, 42 years of age, and presented at the clinic with pain from the hip and thigh while walking. The cup is visibly loose in plain radiography, but is the stem also loose? An ^18^F-fluoride PET/CT scan gave an SUV_max_ of 26 for the cup but only 6 around the stem. At revision surgery, the cup was found to be grossly loose and the stem was well fixed and left in place.

An example of what the ^18^F-fluoride investigation can look like is included for TKA (Fig. 1) and for THA (Fig. 2).

SUV was calculated according to the equation


$${\mathrm{SUV}}_{\mathrm{tissue}}=\frac{\mathrm{BW}*R_{\mathrm{tissue}}}{R_{\mathrm{total}}}$$

where BW equals body weight (in grams), R_tissue_ equals the radioactivity concentration in tissue (in Bq/mL), and R_total_ equals the total injected dose (in Bq). Assuming an average body density of 1 g/mL, this equation gives a unitless value of the regional tissue activity proportional to the average radioactivity concentration in the entire body.

The evaluation was performed with images corrected for attenuation; however, each assessment included an evaluation of potential artifacts in a nonattenuation correction window. The window was set per the uptake of ^18^F-fluoride in each investigation, and it ranged between 5 and 80 in SUV. Each component was assessed in the coronal, transverse, and axial planes for maximum uptake levels at the interface between implant/cement and bone. When the location of the highest periprosthetic uptake value had been identified, a region of interest was created to assess whether higher SUVs were found in any other periprosthetic region. Thus, the area with the maximal ^18^F uptake could be identified, and the obtained SUV was registered as this component’s SUV_max_. High SUVs in the direct vicinity of the joint and surrounding osteophytes were considered remnants of arthritic changes; in this case, they were considered irrelevant and ignored.

### Primary and Secondary Study Outcomes

Our primary study goal was to quantitatively compare the maximal periprosthetic uptake of ^18^F-fluoride around loose versus well-fixed THA and TKA implants in all 63 patients. We thus used the SUV_max_ measuring method to identify a defined point at the interface between implant/cement and bone for each component with maximal ^18^F-fluoride uptake, and then correlated the obtained numeric SUV_max_ with the intraoperative assessment of whether implants were deemed to be loose or well fixed during subsequent revision surgery. We also performed this analysis specific to sex, but no difference emerged between the sexes.

Our secondary goals were to (1) define threshold values for maximum uptake of ^18^F-fluoride indicative of loosening, (2) reassess these threshold values in a subcohort of patients with aseptic but not septic loosening, and (3) investigate whether this method is applicable in everyday practice. To achieve these goals, we used receiver operating characteristic (ROC) curve analysis rendering the area under the curve (AUC) with best thresholds (defined as the SUV_max_ that combined optimal sensitivity and specificity) separately for each component. We then performed a subgroup analysis of patients who were considered not to have an infection according to EBJIS criteria (n = 51). Finally, we compared and analyzed the measurements obtained by two independent observers using Spearman rank correlation and Bland-Altman analysis.

### Study Observation Period

We started our inclusion period in 2016, the time at which the updated PET/CT device became available at our university hospital, well aware of the difficulties in comparing and relating SUV values between different devices. We stopped including patients in 2021 when we performed our analysis.

### Ethical Approval

Ethical approval for this study was obtained from the Regional Ethics Committee in Uppsala, Sweden (2018/338) and from the Swedish Ethical Review Authority (2022-01568-02).

### Statistical Methods

Data distribution was investigated by visual inspection of histograms and quantile-quantile plots and described using mean ± SD or median (IQR), as appropriate. Because SUV results were generally nonnormally distributed, we used the nonparametric Wilcoxon Mann-Whitney U test to compare SUV_max_ values for loose and well-fixed implants. We constructed ROC curves to calculate the AUC as a measure of diagnostic accuracy with 95% confidence intervals (95% CIs). We identified the best thresholds for SUV_max_, differentiating between well-fixed and loose implants using the Youden J statistic, which represents the maximum sum of sensitivity and specificity. We used Spearman rank correlation (ρ) to assess interrater correlations, and we constructed Bland-Altman plots to assess interrater agreement. All statistical analyses were performed using R software (R Core Team; https://www.R-project.org/).

## Results

### Is There a Difference in ^18^F-fluoride Uptake Around Loose Versus Well-fixed THA and TKA Components?

**Fig. 3:**
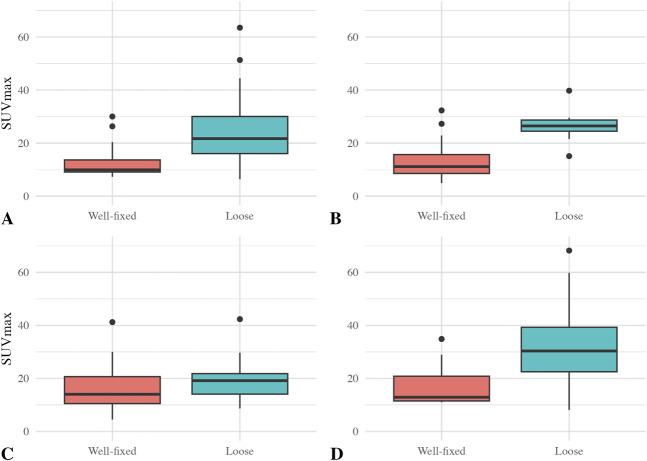
Box plots of the median ^18^F-fluoride SUV_max_ value and IQR for all joints (n = 63) and for (**A**) THA cup (n = 31), (**B**) THA stem (n = 31), (**C**) TKA femoral component (n = 32), and (**D**) TKA tibial component (n = 32) are shown here.

We found higher ^18^F-fluoride uptake around loose versus well-fixed for all arthroplasty components except femoral TKA components. The median (range) SUV_max_ for well-fixed versus loose THA cups was 10 (7 to 30) versus 22 (6 to 64) (difference of medians 12; p = 0.003). The median (range) SUV_max_ for well-fixed versus loose femoral THA stems was 11 (5 to 32) versus 26 (15 to 40) (difference of medians 15; p = 0.001). The median (range) SUV_max_ for well-fixed versus loose tibial TKA components was 13 (11 to 35) versus 30 (8 to 68) (difference of medians 17; p = 0.005) (Fig. 3). In contrast, there was no difference between loose and well-fixed femoral TKA components. The median (range) SUV_max_ for well-fixed versus loose femoral components of TKA was 14 (4 to 41) versus 19 (9 to 42) (difference of medians 5; p = 0.38).

### Can ^18^F-fluoride Uptake Measures Provide a Threshold That Differentiates Loose From Well-fixed Implants Undergoing Revision for a Variety of Septic and Aseptic Indications?

**Fig. 4:**
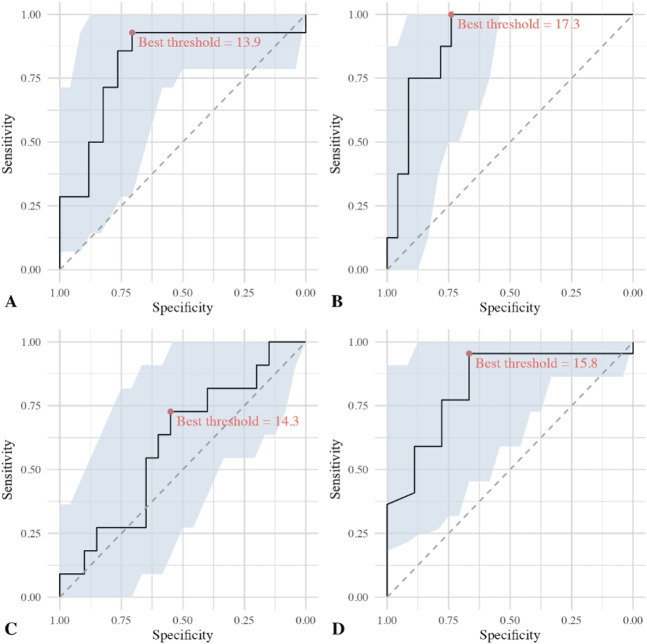
The ROCs and best threshold for the entire study population: (**A**) THA cup (n = 31), (**B**) THA stem (n = 31), (**C**) TKA femoral component (n = 32), and (**D**) TKA tibial component (n = 32) are shown here.

We identified AUCs > 0.8 and useful thresholds distinguishing loose from well-fixed components for all investigated components except femoral TKA components (Table 2). The ROC curve for SUV_max_ around THA cups had an AUC of 0.81 and a best threshold of 13.9, while that around THA femoral stems had an AUC of 0.9 and a best threshold of 17.3. In contrast, the ROC curve for SUV_max_ around femoral TKA components had an AUC of only 0.6 and a best threshold of 14.3, whereas the ROC curve for SUV_max_ around tibial TKA components had an AUC of 0.83 and a best threshold of 15.8 (Fig. 4).

**Table 2.** Test properties for the ability of ^18^F-fluoride PET/CT to detect loosening in all included patients, both aseptic and septic loosening (n = 63)

	AUC (95% CI)	Best threshold^a^	95% CI	Sensitivity (95% CI)	Specificity (95% CI)
THA (cup)	0.81 (0.64-0.98)	13.9	12.5-21.9	0.93 (0.79-1.0)	0.71 (0.47-0.88)
THA (stem)	0.9 (0.79-1.0)	17.3	14.0-24.3	1 (1-1)	0.74 (0.57-0.91)
TKA (femoral component)	0.6 (0.39-0.81)	14.3	8.4-36.3	0.73 (0.45-1.0)	0.55 (0.35-0.75)
TKA (tibial component)	0.83 (0.66-1.0)	15.8	14.0-36.3	0.95 (0.86-1.0)	0.67 (0.33-1.0)

a Best threshold in this ROC curve analysis is the value that maximizes the sum of sensitivity (true positive rate) and specificity (true negative rate) at each specific curve.

### In a Population Restricted to THA and TKA Undergoing Revision for Aseptic Indications, Can Measurement of ^18^F-fluoride Uptake Distinguish Loose From Well-fixed Components?

**Fig. 5:**
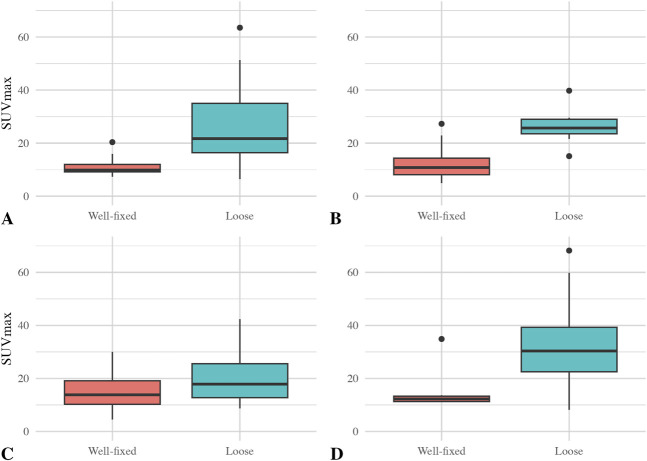
Box plots of the median ^18^F-fluoride SUV_max_ value and IQR for all aseptic joints (n = 51) and for (**A**) THA cup (n = 26), (**B**) THA stem (n = 26), (**C**) TKA femoral component (n = 25), and (**D**) TKA tibial component (n = 25) are shown here.

^18^F-fluoride was even more effective at diagnosing loosening when we limited the population to patients without PJI. The median (range) SUV_max_ for well-fixed versus loose THA cups was 10 (7 to 20) versus 22 (6 to 64) (difference of medians 12; p = 0.001). The median (range) SUV_max_ for well-fixed versus loose femoral THA stems was 11 (5 to 27) versus 26 (15 to 40) (difference of medians 15; p < 0.001). The median (range) SUV_max_ for well-fixed versus loose tibial TKA components was 12 (11 to 35) versus 30 (8 to 68) (difference of medians 18; p = 0.006). There was no difference in SUV_max_ for well-fixed versus loose femoral TKA components of TKA, with a median (range) of 14 (4 to 30) for well-fixed and 18 (9 to 42) for loose femoral TKA components (difference of medians 4; p = 0.24) (Fig. 5).

**Fig. 6:**
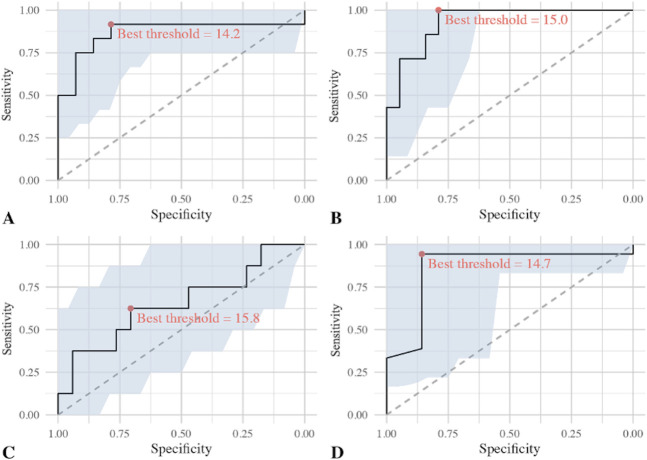
The ROCs and best threshold for the subgroup without suspected or confirmed infection: (**A**) THA cup (n = 26), (**B**) THA stem (n = 26), (**C**) TKA femoral component (n = 25), and (**D**) TKA tibial component (n = 25) are shown here.

The ROC curve analysis for the subgroup of patients without infection indicated an excellent AUC for all but femoral TKA components (Table 3). The ROC curve for SUV_max_ around THA cups had an AUC of 0.87 and a best threshold 14.2. The ROC curve for SUV_max_ around femoral THA stems had an AUC of 0.93 and a best threshold of 15.0. The ROC curve for SUV_max_ around femoral TKA components had an AUC of only 0.65 and a best threshold of 15.8. The ROC curve for SUV_max_ around tibial TKA components had an AUC of 0.86 and a best threshold of 14.7 (Fig. 6).

**Table 3.** Test properties for the ability of ^18^F-fluoride PET/CT to detect loosening in the subgroup restricted to aseptic loosening (n = 51)

	AUC (95% CI)	Best threshold^a^	95% CI	Sensitivity (95% CI)	Specificity (95% CI)
THA (cup)	0.87 (0.7-1.0)	14.2	12.5-21.9	0.92 (0.75-1.0)	0.79 (0.57-1.0)
THA (stem)	0.93 (0.84-1.0)	15.0	14.0-24.3	1 (1-1)	0.79 (0.58-0.95)
TKA (femoral component)	0.65 (0.4-0.91)	15.8	8.5-31.6	0.62 (0.25-0.88)	0.71 (0.47-0.88)
TKA (tibial component)	0.86 (0.67-1.0)	14.7	14.0-18.0	0.95 (0.83-1.0)	0.86 (0.57-1.0)

a Best threshold in this ROC curve analysis is the value that maximizes the sum of sensitivity (true positive rate) and specificity (true negative rate) at each specific curve.

### What Is the Interrater Reliability of Measuring ^18^F-fluoride Uptake?

**Fig. 7:**
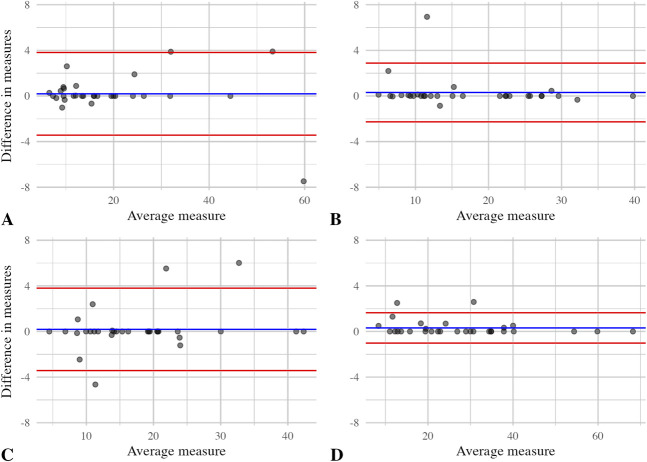
Bland-Altman plots for all investigated components for (**A**) THA cup (n = 31), (**B**) THA stem (n = 31), (**C**) TKA femoral component (n = 32), and (**D**) TKA tibial component (n = 32) are shown here. The blue horizontal lines denote mean values, and the red horizontal lines represent the limits of the 95% CIs.

We found a strong interrater correlation when two independent observers assessed SUV_max_ values, with Spearman ρ ranging from 0.96 to 0.99. Bland-Altman plots showed excellent agreement between the two independent observers (Fig. 7).

## Discussion

The increasing number of arthroplasties worldwide has, expectedly, brought about a large number of patients with painful THA or TKA, where clinical presentation, plain radiography, evaluation of laboratory results, and conventional bone scans alone sometimes do not explain symptoms. Previous investigations indicate that the measurement of ^18^F-fluoride uptake can be helpful in the diagnosis of both aseptic and septic implant loosening, but focus in those studies has been on qualitative rather than quantitative assessments. Our primary aim was thus to evaluate the quantitative analysis of periprosthetic ^18^F-fluoride uptake by ^18^F-fluoride PET/CT to diagnose implant loosening. Our findings suggest that measuring ^18^F-fluoride uptake is a reliable and reproducible method, where the maximum uptake of ^18^F-fluoride, as expressed by SUV_max_, differentiates between loose and well-fixed THA and TKA components with the exception of femoral TKA components. The exclusion of patients with probable or confirmed PJI improved the accuracy of this method. Finally, we established specific threshold values and ratios for SUV_max_ as potential indicators for detecting implant loosening in patients.

### Limitations

This was a retrospective study evaluating a diagnostic test and confirming with surgery. The surgeons were not blinded to the assessment of the ^18^F-fluoride PET/CT and hence could be affected by the preoperative findings during surgery. The reasons for revision varied, and even patients with stable implants were revised for a reason, whether PJI or otherwise. In a cohort of patients who were all scheduled for revision surgery, there is a considerable risk of confirmation bias that could only be eliminated in a prospective study in which surgeons would be blinded to the findings from ^18^F-fluoride PET/CT.

All revision procedures were performed at the same unit, with the participation of several experienced revision surgeons. However, the study’s retrospective design resulted in a lack of a systematic protocol for assessing component stability during surgery. It would be useful to categorize whether implants were grossly loose and came off easily by hand force or whether component removal required substantial force and the use of instruments. Most likely, our interpretation of operative notes was much inferior to such standardized protocols.

The size of our cohort of retrospectively collected patients is restricted, resulting in substantial estimation uncertainty, particularly regarding sensitivity and specificity estimates for threshold values. Connected to this, our failure to find a difference in SUV_max_ around femoral TKA may be related to our limited sample size.

SUV values cannot be directly translated from one device to another, posing a considerable weakness when it comes to real-world applications of our findings. It is, however, technically possible to calibrate PET devices such that SUV_max_ could be considered universal in a similar sense as Hounsfield units are in different CT devices. Nonetheless, the periprosthetic uptake pattern of ^18^F-fluoride, the mostly excellent AUC, and the presence of threshold values are transferable and applicable on similar PET devices.

This study included a number of different implants and different modes of fixation, which probably affected our results because periprosthetic bone metabolism differs between cemented and cementless implants.

The femoral component of TKA is typically manufactured from cobalt/chromium alloys, which could potentially explain the lower accuracy of SUV_max_ measurements around this particular component. This issue has not been specifically studied, but photon attenuation is proportional to the density of the material the photons travel through, and the density of titanium (4.5 g/mL) is substantially lower than that of cobalt (8.9 g/mL) and chromium (7.2 g/mL). The impact of prosthesis materials on the possibility of detecting loosening by the use of metabolic imaging must therefore be studied further. In addition to its composition, the shape of the femoral component, strikingly like a bowl, most likely affects PET measurements. The decay of the tracer and the ability of the PET device to simultaneously detect two photons emerging at an angle of 180° from bone that is partly hidden by the implant may be more obstructed by femoral TKA components.

Including patients both with and without PJI in our study was a potential limitation. Thus, to disentangle the potential bias introduced by mixing patients with and without PJI, we performed a sensitivity analysis after exclusion of patients with infections. The exclusion of patients with PJI improved the accuracy of the investigated method, thereby supporting the validity of our findings for patients without PJI. However, differentiation between patients with and without infections is not always easy in clinical practice. Furthermore, there is a risk of false-negatives in our study, in that patients with low-grade PJI may have had negative tissue cultures at the time of revision surgery and were thus categorized as noninfected but returned later with a fully manifest PJI. Nonetheless, including patients with potential but not fulminant PJIs in our cohort can guide physicians who use ^18^F-fluoride PET/CT in the diagnostic assessment of patients prior to revision surgery.

The SUV_max_ measurements in our study were performed by inexperienced PET/CT observers. They did receive training by an expert nuclear physician, but their status as novices in the field of nuclear medicine obviously represents a limitation to our results. However, our evaluation showed excellent reliability and represents a possible way of interpretation that, with rather small effort, can be taught to arthroplasty surgeons.

Another limitation of this study is the varying time between the ^18^F-fluoride PET/CT and the subsequent revision procedure (in one patient, 3 years had passed between the ^18^F-fluoride PET/CT and revision surgery). This time interval limits the value of the comparison because an aseptic loosening may have progressed into an infection because of hematogenous bacterial spread. However, we have no indications of this in any of our patients.

Finally, the varying time span between the index arthroplasty procedure and the ^18^F-fluoride PET/CT investigation was an issue in our study. Two of the investigations in our study were performed less than a year from the index procedure, which can result in postoperatively enhanced bone metabolism and distorted SUV_max_.

### Is There a Difference in ^18^F-Fluoride Uptake Around Loose Versus Well-fixed THA or TKA Components?

We found higher ^18^F uptake around loose versus well-fixed components for all but the femoral TKA components. This is of clinical use in the evaluation and diagnostic assessment prior to revision surgery when encountering nonconclusive findings in patients with painful THA or TKA. Only a few studies have focused on the use of ^18^F-fluoride PET/CT to diagnose aseptic implant loosening. In one of the studies on this issue, Koob et al. [16] presented a retrospective study of 26 patients with 37 implants, of whom 18 were revised and 11 were considered “clinically silent.” No SUVs were measured; instead, a qualitative description of the uptake pattern was applied to determine whether implants were loose. However, the authors reported an overall sensitivity of 95% and a specificity of 87%.

A drawback in SUV measurement is that the results depend on image reconstruction settings and scanner resolution and do not directly transfer from one PET/CT device to another.

A noteworthy finding is that the femoral TKA component seems to behave differently compared with the other investigated components and seems to be challenging to evaluate by the use of ^18^F-fluoride PET/CT. The underlying cause for this different behavior is unknown and warrants further inquiry. One theory is related to different biomechanical load distributions, which could cause higher and more unspecific uptake underneath the femoral shield. Duus et al. [8] investigated 41 patients with bone scanning after TKA and found a prolonged uptake up to 2 years after surgery, possibly supporting this theory. In line with that finding, a study by Son et al. [29] found that the uptake pattern of ^18^F-fluoride in asymptomatic joint reconstructions differed between THA and TKA. TKA showed a prolonged curvilinear uptake pattern with an increase up to 15 months after surgery, as compared with THA. This could be another reason for the poor accuracy for the femoral component of TKA. Another reason could be that the femoral component absorbs more photons and induces more severe SUV artifacts when attenuation correction based on CT is performed.

### Can ^18^F-fluoride Uptake Measures Provide a Threshold That Differentiates Loose From Well-fixed Implants Undergoing Revision for a Variety of Septic and Aseptic Indications?

We found ROC curves with good to excellent AUC when differentiating loose from well-fixed arthroplasty components except for femoral TKA components. Even in a mixed material with both aseptic and septic loosenings, the diagnostic accuracy of this method was high.

Kobayashi et al. [15] also calculated ROC curves, and a threshold for infection was set at an SUV_max_ of 6.9 with 81% sensitivity and 80% specificity (AUC 0.87). Their threshold for aseptic loosening was 4.9, with 95% sensitivity and 82% specificity (AUC 0.96). In both our ROC analysis and theirs, the levels of sensitivity and specificity render a high degree of diagnostic accuracy. In contrast to those authors, our thresholds for aseptic loosening were between an SUV_max_ of 14 to 20, but such values must be calibrated individually for each PET/CT device.

### In a Population Restricted to THA and TKA Undergoing Revision for Aseptic Indications, Can Measurement of ^18^F-fluoride Uptake Distinguish Loose From Well-fixed Components?

^18^F-fluoride PET/CT was also very effective at diagnosing loosening when we limited the population to those patients believed not to have PJI. The difference in uptake between loose and well-fixed components persisted, and when a threshold was set at an SUV_max_ of 15 on our device, most aseptic loosenings were correctly diagnosed.

Kobayashi et al. [15] used ^18^F-fluoride PET to differentiate septic from aseptic loosening in patients who had undergone THA. Some 65 joints were investigated, but only 27 were surgically revised. Eleven patients were treated nonsurgically for suspicion of aseptic loosening after THA, and another 27 were derived from an asymptomatic control group. The authors identified significant differences in SUV_max_ uptake between the control and aseptic group as well as between the aseptic and septic groups, which is in line with our findings.

Kumar et al. [18] established the superiority of ^18^F-fluorodeoxyglucose (^18^F-FDG) PET/CT over ^18^F-fluoride NaF PET/CT in the diagnosis of PJI. Several other groups have presented findings that indicate an increased risk for false-positive results with ^18^F-FDG [14, 19, 36, 37]. Taking our results into account, we suggest that ^18^F-fluoride and ^18^F-FDG could be combined in patients with suspected PJI.

### What Is the Interrater Reliability of Measuring ^18^F-fluoride Uptake?

Interrater reliability was high for the measurements of ^18^F uptake despite the fact that determination of SUV_max_ can be challenging. There is a multitude of potential errors, not only in specifying the relevant uptake amount but also concerning attenuation correction. Although the observers in our study had different professional positions (orthopaedic surgeon versus medical student), the correlation between these two observers was excellent after careful instruction by a nuclear physician. Quantitative analysis is nearly always more reproducible than qualitative analysis. Therefore, focusing on the overall SUV_max_ around the implant instead of different zones might be a more straightforward approach for evaluating implant stability.

### Conclusion

Measuring SUV_max_ after ^18^F-fluoride PET/CT is a useful adjunct in the diagnostic evaluation of suspected implant loosening after THA and TKA. The method appears to be both accurate and reliable in diagnosing implant loosening for all investigated components—except femoral TKA components. In a real-world scenario including patients with both low-grade infections and aseptic loosening, the investigated method seems to be both applicable and fairly easy to learn. When PJI can be ruled out by other means, measurements of ^18^F-fluoride uptake by PET/CT most likely perform even better, as indicated by the larger AUCs in the subgroup analysis of patients without infection. Further prospective studies are warranted, not least to explore the reasons behind the much poorer diagnostic accuracy of femoral TKA component loosening. We suggest that future studies be prospective in their design, with a structured study protocol for the surgical evaluation of implant fixation during revision surgery and a follow-up of no less than 2 years after the index procedure.
